# Theory of Hydrogen Migration in Organic–Inorganic Halide Perovskites**

**DOI:** 10.1002/anie.201502544

**Published:** 2015-06-12

**Authors:** David A Egger, Leeor Kronik, Andrew M Rappe

**Affiliations:** Department of Materials and Interfaces, Weizmann Institute of Science Rehovoth 76100 (Israel) E-mail: david.egger@weizmann.ac.il leeor.kronik@weizmann.ac.il; The Makineni Theoretical Laboratories, Department of Chemistry, University of Pennsylvania Philadelphia, PA 19104-6323 (USA) E-mail: rappe@sas.upenn.edu

**Keywords:** density functional calculations, hybrid perovskites, proton transport, solar cells

## Abstract

Solar cells based on organic–inorganic halide perovskites have recently been proven to be remarkably efficient. However, they exhibit hysteresis in their current–voltage curves, and their stability in the presence of water is problematic. Both issues are possibly related to a diffusion of defects in the perovskite material. By using first-principles calculations based on density functional theory, we study the properties of an important defect in hybrid perovskites—interstitial hydrogen. We show that differently charged defects occupy different crystal sites, which may allow for ionization-enhanced defect migration following the Bourgoin–Corbett mechanism. Our analysis highlights the structural flexibility of organic–inorganic perovskites: successive iodide displacements, combined with hydrogen bonding, enable proton diffusion with low migration barriers. These findings indicate that hydrogen defects can be mobile and thus highly relevant for the performance of perovskite solar cells.

Organic–inorganic halide perovskites (OIHPs) are an intriguing class of materials, in which the A site of a cubic perovskite material ABX_3_ is an organic molecule, B is typically a divalent metal, and X a halide ion.[1] In recent years, OIHPs have emerged as promising candidates for photovoltaic applications.[2–8] Power-conversion efficiencies have improved at a record speed and now approach 20 %.[9, 10] Despite the remarkable technological progress, a microscopic picture of the inner workings of hybrid perovskite solar cells is incomplete, due to a dearth of fundamental insight into the role of the OIHP absorber. Specifically, mechanisms that explain the hysteresis observed in current–voltage curves of OIHP-based cells,[11, 12] as well as the general stability of these materials, are among the major scientific challenges at present.[13–15]

The possibility of ionic diffusion is highly relevant for both these challenges. First, the migration of charged defects (among other effects) has been hypothesized, on the basis of impedance spectroscopy measurements[16] and switchable photocurrent effects,[17] to play an important role in the above-mentioned hysteresis phenomenon.[11, 12] Second, ion diffusion is well-known to play an important role in material stability (or lack thereof) in inorganic thin-film solar-cell materials.[18, 19]

Herein, we take a first step toward understanding ionic migration in OIHP materials, by studying the migration of interstitial hydrogen from first principles. Our interest in hydrogen motion is stimulated by the well-known proton-conducting phenomena in oxide perovskites,[20, 21] which could also be relevant for OIHPs. Indeed, hydrogen is a pertinent defect species in both prototypical organic[22, 23] and inorganic semiconductors,[24, 25] in part because it is ubiquitous in many processes of organic and inorganic chemistry. Specifically, water is a solvent for lead-based OIHPs. Recent experiments have demonstrated the beneficial effect of mild moisture exposure on film growth,[26] and it was suggested that (partial) decomposition of OIHPs as a result of water may trigger a proton-migration process.[27] Furthermore, the organic cation could be deprotonated, thus indicating a possible intrinsic source of migrating hydrogen. We, therefore, explore the possibility of mobile hydrogenic defects in OIHPs.

To explore the driving forces for interstitial hydrogen defects in OIHPs we first calculated the adiabatic potential energy surface (PES) for H^+^, H^0^, and H^−^ in a 2×2×1 supercell (16 formula units) of tetragonal methylammonium lead iodide (MAPbI_3_, see Figure 1 a; see the Theoretical Methods Section and Supporting Information for complete details). Hydrogen migration will be accompanied by partial or complete lattice relaxation, depending on the time scales involved.[28] Therefore, we consider two extreme cases. In one, we do not allow for any lattice relaxation and consider the potential landscape on the plane defined in Figure 1 b. In the other, we allow for full relaxation by optimizing all the ionic coordinates, including sampling of the migration path.

**Figure 1:**
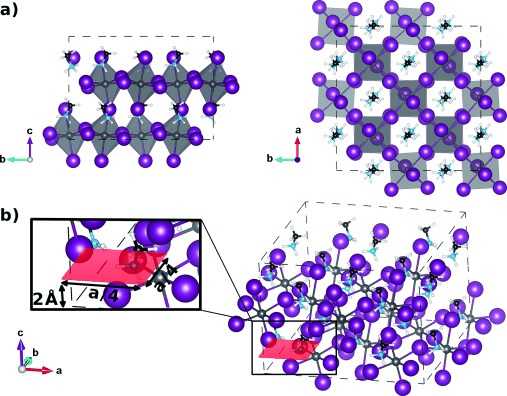
a) Front (left) and top (right) view of the 2×2×1 supercell of the tetragonal phase of methylammonium lead iodide (MAPbI_3_). This supercell contains 16 formula units of MAPbI_3_ and allows for the inclusion of distorted, tilted, and rotated octahedra. b) Sketch and zoom of a cross-sectional plane in the unit cell (shown in red), chosen to sample the energy landscape of hydrogenic defects in MAPbI_3_ without lattice relaxation. The dashed lines indicate the supercell, containing carbon (black), nitrogen (blue), hydrogen (white), iodine (violet), and lead (light gray) atoms; in (a), the latter are inside the shaded octahedra. For convenient visualization, some atoms in adjacent unit cells are shown.

The adiabatic (i.e., frozen lattice) PES and schematic structural representations of the minimum-energy locations of the H^+^, H^−^, and H^0^ impurity in MAPbI_3_ are shown in Figure 2 a,b, respectively. All three hydrogenic defects are found to be repelled from the A site, as MA is a rather large ion with six hydrogen atoms that interact repulsively with impurities. However, the positively charged H^+^ ion (left panel) is stable in the vicinity of the anions (i.e. iodides), the charge-neutral H^0^ species (center panel) is in an interstitial lead iodide site, and the negatively charged H^−^ ion (right panel) is closer to the B site, namely, to the Pb cation. Following previous findings as to hydrogen defects in GaN,[24] these dissimilar preferences of the differently charged impurities can be understood by recalling the partially ionic character of MAPbI_3_: The positive H^+^ ion is attracted to the anionic iodide, where it is screened from the cationic MA and Pb. Conversely, the H^−^ ion is attracted to a cation. Indeed, this rationale is strongly reflected in the results of Figure 2, which reveal rather short distances of 1.7 Å for I-H^+^, 2.0 Å and 2.1 Å for I-H^0^ and Pb-H^0^, respectively, and 1.9 Å for Pb-H^−^. Interestingly, the bonding of the neutral H^0^ species is determined by covalent interactions with both Pb and I, as confirmed by the projected local density of states (see Figure S1 in the Supporting Information). H^0^ is thus stable at sites that are essentially in between the preferred sites of H^+^ and H^−^.

**Figure 2:**
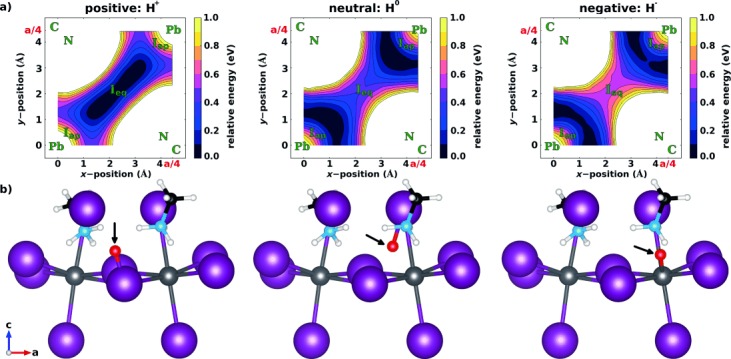
a) Contour plots of the adiabatic, “frozen lattice” potential energy surface, in the cross-sectional plane shown in Figure 1 b, for the interstitial defects H^+^ (left), H^0^ (center), and H^−^ (right) in MAPbI_3_. Green letters indicate the sites of neighboring non-hydrogen atoms (“eq” and “ap” subscripts denote equatorial and apical iodide sites, respectively) and isolines are drawn at energy differences of 0.1 eV. b) Fully optimized positions of H^+^ (left), H^0^ (center), and H^−^ (right) in the otherwise unrelaxed MAPbI_3_ lattice. For easy visualization, the interstitial hydrogenic defect is enlarged, red, and marked by an arrow.

We now consider the case where all the ions are fully relaxed in response to the hydrogen impurity. The above-discussed electrostatic and covalent effects are the main driving forces for motion of a hydrogen impurity, even when lattice relaxation is included (see Figure S2 in the Supporting Information). Indeed, the distances between each hydrogen impurity and its nearest ion change very little (0.02–0.07 Å). However, significant additional lattice relaxations occur for the charged defects (see Figure S2 in the Supporting Information). Interestingly, an H^+^ ion allows the iodides to move closer together, so that it can bridge two iodide sites in MAPbI_3_ (analogous behavior has been discussed in oxide perovskites).[20, 21, 29] Depending on the lattice site, the resulting iodide rearrangements can be significant: the distances of neighboring iodides are reduced by up to 0.6 Å, accompanied by octahedral rotations of up to about 4°. Furthermore, MA molecules close to H^+^ ions rotate such that repulsive interactions of the defect and the rearranging iodides with the MA cation are reduced. For the hydride, full structural relaxation shows that the H^−^ impurity moves approximately 0.4 Å closer to the ammonium group and that the ammonium–Pb distance is also reduced.

These findings depict the interactions between charged defects and host constituents, which are important in general, but particularly since these may affect the stability and structural order of OIHPs. Here, we emphasize the internal structural flexibility of hybrid perovskites, namely rather weakly bound X sites rearranging with little energy cost, and an organic molecule that rotates relatively easily. Both effects can be beneficial for (charged) defect stabilization.

Our results for the structure and energetics of hydrogenic impurities suggest the possibility of an ionization-enhanced hydrogen migration in hybrid perovskites, following the Bourgoin–Corbett mechanism,[30] which has been discussed previously for inorganic semiconductors such as Si.[31, 32] In this mechanism, the interstitial H^+^ impurity can capture one (or two) athermal electrons, which are present in abundance in the operational, illuminated solar cell, and turn into H^0^ (or H^−^). It can then move to lower energy by migrating from the iodide to an interstitial lead iodide (or lead) site. The same reasoning holds for H^0^ and H^−^, which upon capturing electrons/holes, that is, enhanced through ionization processes, will migrate to a different site in the lattice to minimize the total energy. With the aid of an external electric field, this can result in a net migration of defects. Quantitatively, the relative importance of this mechanism, variations of which may also be relevant for other types of defects or hosts, would naturally depend on defect formation energies, doping levels, and behavior under illumination.

Finally, having discussed the possible effects of the charge state, we now focus on thermally activated diffusion of H^+^, which is the smallest species and one whose migration has been studied in other perovskites.[20, 21, 28, 29, 33] Specifically, we examine the minimum energy path (MEP) and (classical) migration barrier for proton migration between equatorial iodides in MAPbI_3_. From its local minimum position, H^+^ migrates along a transient hydrogen bond connecting two equatorial iodides (see Figure 3 b), both of which rearrange significantly during the process, as expected from the full structural relaxation for the case of H^+^ discussed above. This is followed by a rotational over-barrier motion of the proton closely attached to the iodide (Figure 3 b), after which H^+^ stabilizes in a local minimum, bridging the equatorial and apical iodide. This local structural minimum again benefits from significant rearrangements of the iodide atoms, which means that overall proton migration is enhanced by successive displacements of iodide atoms. Similar processes have indeed been described in oxide perovskite proton conductors.[20, 21, 28, 29, 33] The MEP-calculated energy barrier for lateral iodide-to-iodide proton migration is 0.29 eV (see Figure 3 a), which is relatively low. Additional MEP calculations (see Figure S3 in the Supporting Information) indicate that energy barriers for iodide–iodide H^+^ transfer can even be as low as 0.17 eV. If proton migration is fast enough to preclude lattice relaxation, in particular iodide rearrangements, then H^+^ has to overcome larger distances between two iodide sites. The migration barrier for a proton then increases to 0.4–0.5 eV (see Figure S4 in the Supporting Information), in good agreement with barriers gleaned from the low-energy channel in the PES of H^+^ given in Figure 2 a. Nuclear quantum effects such as tunneling were not considered in our classical MEP calculations, but will likely further reduce the migration barrier,[34–36] thus making H^+^ transfer even faster. The largely unknown properties of MAPbI_3_ under operating conditions, such as effective defect concentrations as well as light- and temperature-induced effects, will likely play an important role in understanding which of the defect-migration mechanisms here discussed will be most relevant under various practical conditions. Overall, the calculated migration barriers are comparable to those previously calculated for oxide perovskite proton conductors,[28, 29, 33] which is reasonable given the similar migration mechanism and suggests the possibility of mobile hydrogen impurities in MAPbI_3_.

**Figure 3:**
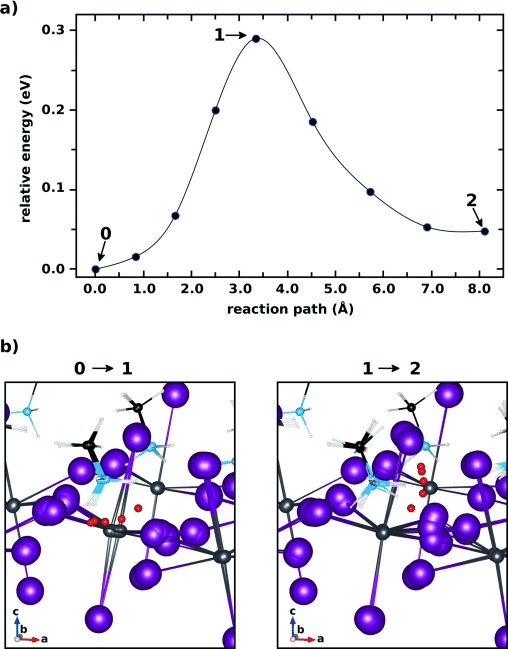
a) A minimum energy path (MEP) for H^+^ in MAPbI_3_ connecting two equatorial iodide sites, obtained with full relaxation of the surrounding lattice. The blue circles represent the calculated total energies of the images used to sample the MEP and the thin black line is a cubic-spline interpolation that serves as a guide to the eye. b) Visualization of the nuclear motions associated with the MEP shown in (a): superposition of the geometries used to sample section “0” to “1” (left panel) and section “1” to “2” in the MEP (right panel). For easy visualization, H^+^ is colored in red and shown larger. Note the significant rearrangement of iodide, lead, and MA molecules close to H^+^.

In summary, we have provided a first-principles study of the energetics and structural properties of hydrogenic interstitial impurities in the organic–inorganic halide perovskite MAPbI_3_. On the basis of density functional theory calculations, it was shown that the charge-density distribution in hybrid perovskites results in distinctly different local minimum sites for differently charged hydrogen defects. This allows for ionization-enhanced migration of hydrogen impurities in hybrid perovskites, which may also be relevant for other types of defects in OIHPs. Furthermore, we examined structural relaxations induced by hydrogen impurities and found that these can be significant, especially for charged defects. Specifically, the structural flexibility of hybrid perovskites allows for collective iodide displacements that can enhance proton diffusion through MAPbI_3_. The calculated migration barriers for proton transfer are relatively low, from which we conclude that protons are likely to be mobile under device-relevant conditions in MAPbI_3_. The computational results presented here suggest that migration of hydrogen-like defects, introduced either extrinsically or intrinsically, may play an important role in understanding the microscopic origins of hysteresis effects and stability-related issues in hybrid perovskite materials. We hope that our theoretical assessment will prompt further experimental efforts aimed at investigating this issue.

## Theoretical Methods

We performed density functional theory (DFT) calculations using the VASP code.[37] The exchange-correlation energy was described using the Perdew–Burke–Ernzerhof (PBE)[38] form of the generalized-gradient approximation, augmented by Tkatchenko–Scheffler pair-wise terms[39] that incorporate dispersive interactions, already shown to be important for calculations of OIHPs[40, 41] and defect diffusion in semiconductors.[42] To calculate minimum energy paths and (classical) migration barriers, nudged elastic band (NEB) calculations were performed using the VTST extension of the VASP code, where we employed the climbing-image technique.[43, 44] We note that defect energetics are generally sensitive to the choice of exchange-correlation functional.[45, 46] In the Supporting Information, we provide additional approximate MEP calculations based on using a hybrid functional or the self-consistent screening procedure[47] for obtaining dispersive corrections (see Figure S5) and show that these additions do not change our main conclusions. Schematic representations of the crystal structures were generated using the VESTA program.[48] See the Supporting Information for full technical details.

## Supporting Information

As a service to our authors and readers, this journal provides supporting information supplied by the authors. Such materials are peer reviewed and may be re-organized for online delivery, but are not copy-edited or typeset. Technical support issues arising from supporting information (other than missing files) should be addressed to the authors.

miscellaneous_information
