# Supplementary material for: Theory of Hydrogen Migration in Organic–Inorganic Halide Perovskites
Source: Angew Chem Int Ed Engl. 2015 Jun 12;54(42):12437–41. doi: 10.1002/anie.201502544 (PMC4643191; doi:10.1002/anie.201502544)
Supplement: Supplementary file 1 — miscellaneous_information [file anie0054-12437-sd1.pdf]

Angewandte Chemie
Eine Zeitschrift der Gesellschaft Deutscher Chemiker

Supporting Information

Theory of Hydrogen Migration in Organic–Inorganic Halide Perovskites**

David A. Egger, Leeor Kronik,* and Andrew M. Rappe**

anie_201502544_sm_miscellaneous_information.pdf

Contents

I. Technical Details of the Calculations and System Setup S2

II. Additional Calculations and Results S5

A. Electronic Structure of Charge-Neutral H^0 in $MAPbI_3$ S5

B. Comparison of Partially and Fully Relaxed Structures of Hydrogen Impurities in $MAPbI_3$ S6

C. Additional Minimum Energy Path of a Protonic Impurity in $MAPbI_3$ including Relaxation S7

D. Barrier for Migration of a Protonic Impurity in $MAPbI_3$ without including Relaxation S8

E. Approximate Minimum Energy Path Calculations using a Hybrid Functional and Many-body Dispersive Corrections S9

I. Technical Details of the Calculations and System Setup

We performed density functional theory (DFT) calculations using the VASP code.[1] Convergence criteria of 10^{-6} eV per unit cell for the total energy in the electronic self-consistent cycle, and 5×10^{-3} eV/Å for minimizing the forces on the atoms when calculating local structural minima, were employed. A planewave basis (kinetic-energy cutoff: 400 eV) was used, with a 1×1×2 *k*-point grid sampling. This is a rather sparse grid, but is still appropriate as we have been using a relatively large super cell in all our calculations (*vide infra*). Furthermore, the projector-augmented wave (PAW) formalism[2] was used to treat core-valence electron interactions. Specifically, we chose the 'normal' (*i.e.*, not 'soft'), program-supplied PAW potentials, and treated the 5*d* electrons of Pb explicitly. Using 'hard' PAW potentials and increasing the cutoff energy to 700 eV changed the optimized I-H^+ distance in the local minimum geometry by less than 0.001 Å.

Nudged elastic band (NEB) calculations have been performed with the VTST extension of the VASP code, where we employ the climbing-image technique.[3,4] We sampled each defect migration path with seven images, and optimized the forces on the images until they dropped below 10^{-2} eV/Å.

Exchange and correlation were described using the Perdew-Burke-Ernzerhof (PBE) functional.[5] Some of us have previously shown that spin-orbit coupling (SOC) affects the structural properties of OIHPs only to a limited extent.[6] Since here we are studying hydrogen migration by calculating structural properties and mapping the energy landscape of defect geometries, we do not account for SOC in our calculations. The PBE-predicted bandgap calculated without considering SOC is then found to be in good agreement with experiment.[7] This agreement is, however, entirely coincidental because the error in underestimating the

gap, as expected in Kohn-Sham DFT in general,[8,9] fortuitously cancels the error introduced when neglecting SOC. More importantly for our present purposes, PBE is known to typically describe shorter covalent and ionic bonds sufficiently accurately, but fails to capture long-range dispersive interactions that are crucial in OIHPs.[10,11] Therefore, as mentioned in the main text, we followed our previous strategy[6] and added pair-wise dispersion interactions, computed using the planewave implementation[12,13] of the Tkatchenko-Scheffler (TS) pair-wise dispersion scheme,[14] to the PBE results.

To calculate the cross-sectional plane of the potential energy surface (PES), we sampled a slice in the ab-plane of the unit-cell of $MAPbI_3$, defined in Figure 1b of the main text, with a uniformly-spaced grid of 100 points, and used the grid-points as defect sites in a series of total energy DFT calculations. The final PES was then computed from these total energies using a cubic-spline interpolation, and aligned to the minimum energy. Regions of relative energies that are >1 eV compared to the respective minimum energy are shown in white in Figure 2a in the main text.

It is important to note that depending on the properties of the host material, in particular the position of the Fermi level and concentration of defects, hydrogen that is incorporated as an impurity can exhibit differently charged states, i.e., that of a proton (H^+), neutral hydrogen atom (H^0), and hydride ion (H^-). In the absence of detailed information concerning these aspects of OIHPs, we considered all three hydrogen-like defects in methylammonium-lead-iodide ($MAPbI_3$). To this end, the number of electrons present in the unit-cell, N_e, was constrained to $N_e = N_p - x$, where N_p is the number of protons per cell, and x was set to +1, 0, -1, and 0 for modeling H^+, H^0, and H^-, respectively. Note that for H^+ and H^- this necessitates a compensating uniform background charge in the calculations. To minimize artificial interaction among defects in different unit cells, one H-like defect has been

incorporated in a 2x2x1 super-cell of the tetragonal phase of $MAPbI_3$ (see Figure 1a in the main text), i.e., we also consider the octahedral distortions in our calculations. This super-cell contains 16 formula units of $MAPbI_3$, and is thus 16 times larger than the primitive unit cell of $MAPbI_3$.

II. <u>Additional Calculations and Results</u>

A. Electronic Structure of Charge-Neutral H^0 in $MAPbI_3$

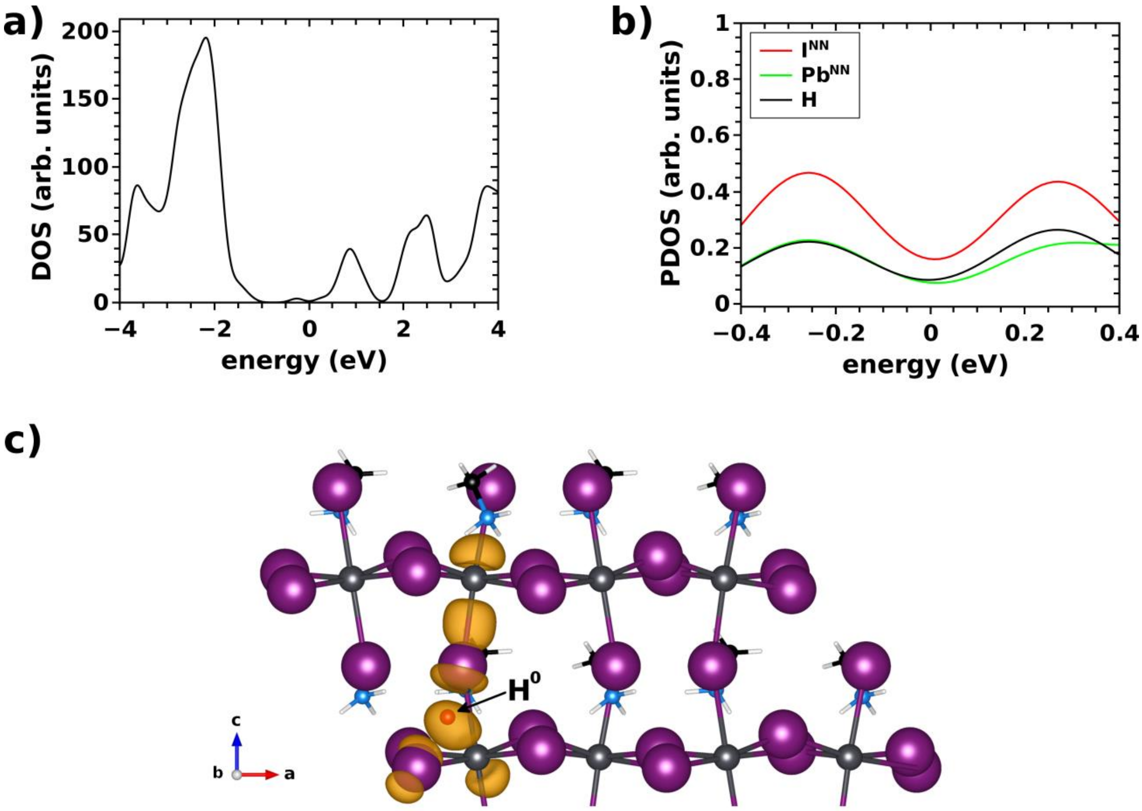

Figure S1: a) Density of states (DOS) of methylammonium-lead-iodide ($MAPbI_3$) with one interstitial neutral H defect present in the unrelaxed 2x2x1 super-cell of $MAPbI_3$ (see Figure 2b, center part, in the main text). b) Projected DOS (PDOS) associated with the H defect and the nearest neighbor iodide (I^{NN}) and lead (Pb^{NN}) atoms. c) Local DOS (LDOS), shown as an isosurface plot in orange, obtained by integrating in an energy-window of ±0.1 eV around the maximum of the lower-energy peak in the PDOS. The isovalue is set to 0.02 $–e\text{Å}^{-3}$ and the position of the H^0 defect is highlighted by an arrow. These results indicate the covalent character of the interactions that determine the energetics and local minimum position of the H^0 defect.

B. Comparison of Partially and Fully Relaxed Structures of Hydrogen Impurities in $MAPbI_3$

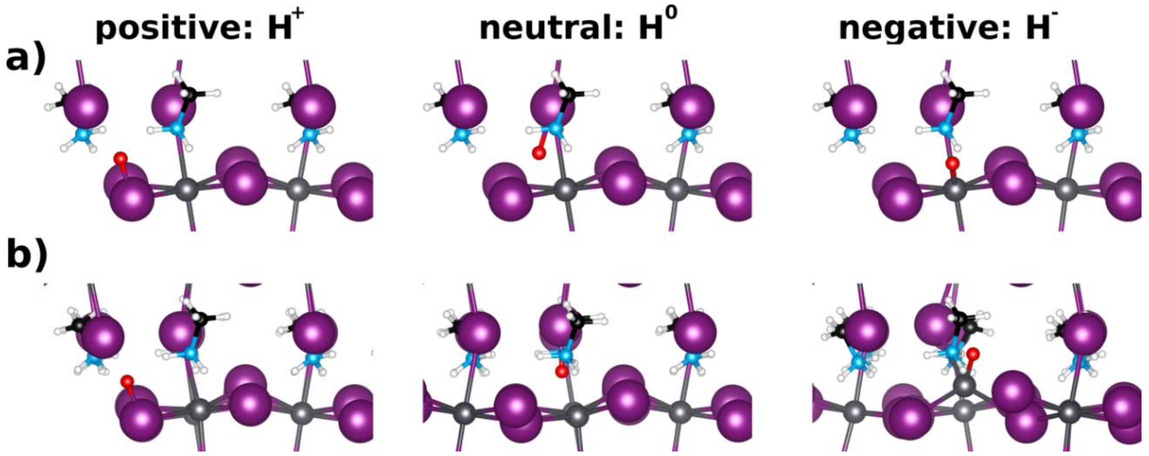

Figure S2: a) Fully-optimized positions of H^+ (left), H^0 (center), and H^- (right) in the otherwise unrelaxed $MAPbI_3$ lattice. b) The same as in part a, but including lattice relaxations. For easy visualization, the interstitial hydrogen-like defect is larger and colored in red. Relaxations do not alter the driving forces and minimum position of the hydrogenic defects, but for the charged hydrogen impurities, significant relaxations of the surroundings also occur.

C. Additional Minimum Energy Path of a Protonic Impurity in $MAPbI_3$ including Relaxation

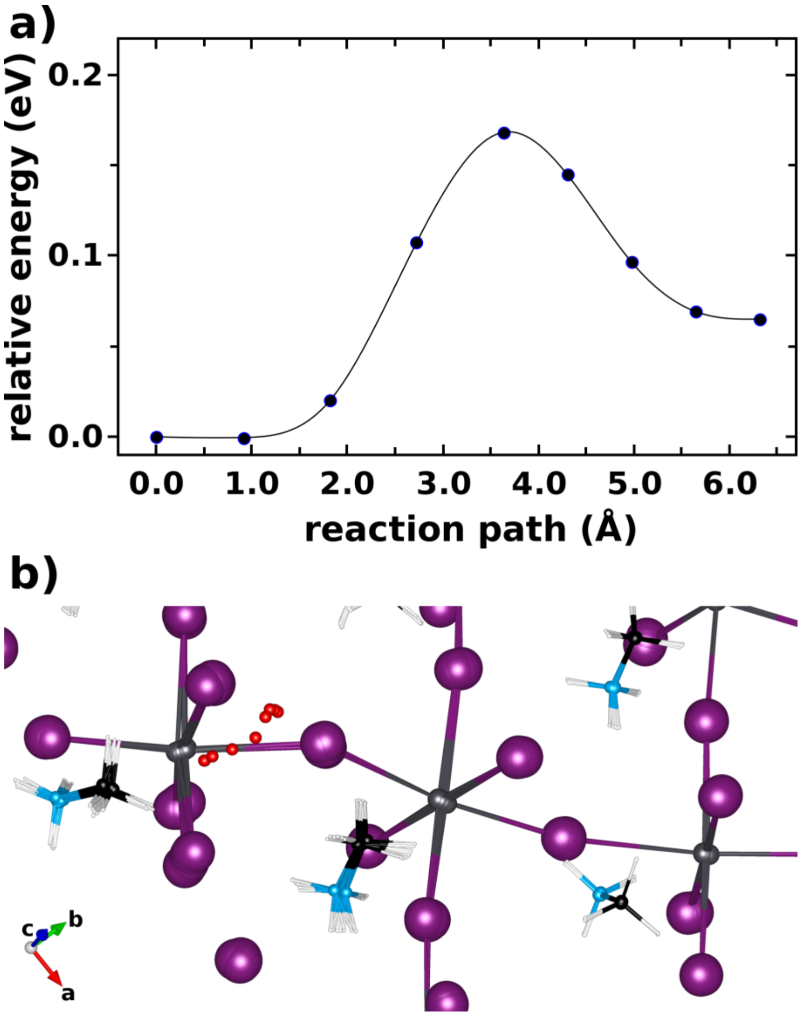

Figure S3: a) A minimum energy path (MEP) including relaxation for H^+ in $MAPbI_3$ connecting two different iodide sites. Blue discs represent total energies of the images used to sample the MEP, and the thin black line is a cubic-spline interpolation that serves as a guide to the eye. The energy barrier is calculated to be 0.17 eV. b) Superposition of the geometries that correspond to the MEP shown in part a. Note the significant rearrangement of ions.

D. Barrier for Migration of a Protonic Impurity in $MAPbI_3$ without including Relaxation

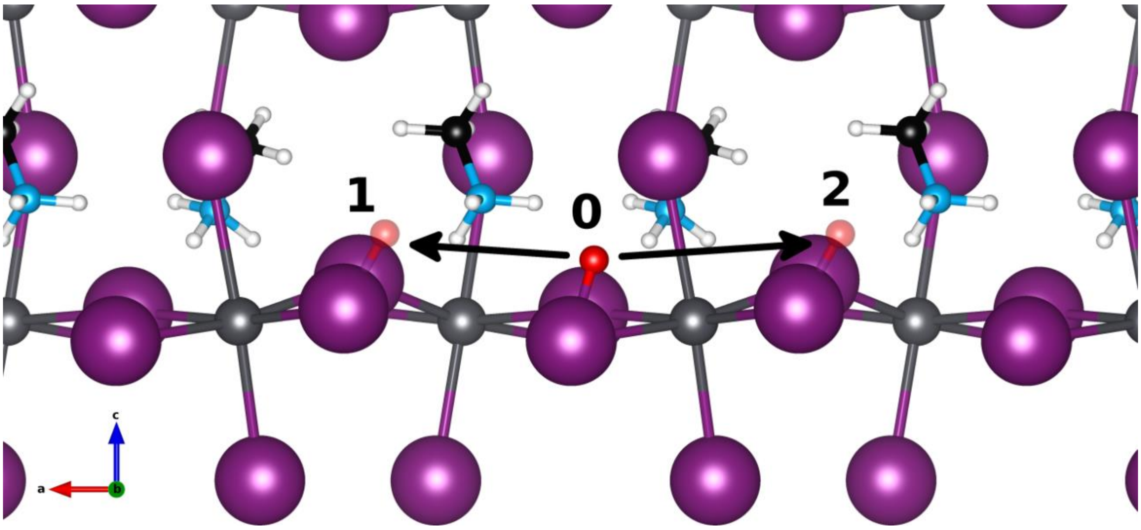

Figure S4: Sketch of the energy barrier for two possibilities of H^+ migration in $MAPbI_3$ without considering relaxation. The energy barrier from “0” to “1” is calculated to be 0.46 eV and from “0” to “2” 0.40 eV. These are relatively low, but larger than those obtained when including lattice relaxations (see main text).

E. Approximate Minimum Energy Path Calculations using a Hybrid Functional or Many-body Dispersive Corrections

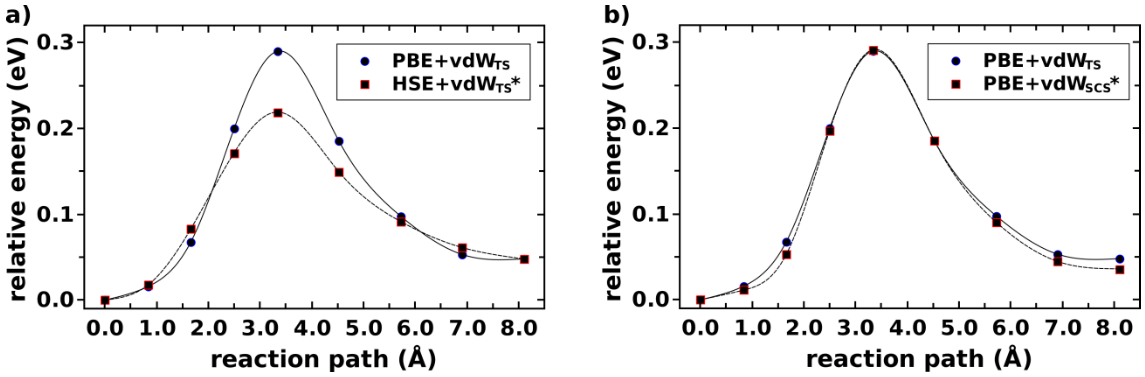

Figure S5: The minimum energy path (MEP) for H^+ in MAPbI3 connecting the two equatorial iodide sites, as calculated in Figure 3 of the main text (method denoted as PBE+vdW$_{TS}$ in the figure), recomputed with the HSE functional[15,16] (HSE+vdW$_{TS}$*, part a) or using the self-consistent screening procedure for obtaining dispersive corrections[17] (PBE+vdW$_{SCS}$*, part b). Due to the computational expense associated with these methods, we have used the PBE+vdW$_{TS}$ geometries throughout. We note that the images used to sample the HSE+vdW$_{TS}$ and PBE+vdW$_{SCS}$ MEPs are therefore only valid approximately (as emphasized by the '*' superscript). Compared to results from PBE+vdW$_{TS}$, the migration barrier of H^+ is either slightly lowered or very similar when using HSE+vdW$_{TS}$* or PBE+vdW$_{SCS}$*.

References

[1] G. Kresse, J. Furthmüller, *Phys. Rev. B* **1996**, *54*, 11169–11186.
[2] G. Kresse, D. Joubert, *Phys. Rev. B* **1999**, *59*, 1758–1775.
[3] G. Henkelman, B. P. Uberuaga, H. Jónsson, *J. Chem. Phys.* **2000**, *113*, 9901–9904.
[4] G. Henkelman, H. Jónsson, *J. Chem. Phys.* **2000**, *113*, 9978.
[5] J. P. Perdew, K. Burke, M. Ernzerhof, *Phys. Rev. Lett.* **1996**, *77*, 3865–3868.
[6] D. A. Egger, L. Kronik, *J. Phys. Chem. Lett.* **2014**, *5*, 2728–2733.
[7] J. Even, L. Pedesseau, J.-M. Jancu, C. Katan, *J. Phys. Chem. Lett.* **2013**, *4*, 2999–3005.
[8] J. P. Perdew, M. Levy, *Phys. Rev. Lett.* **1983**, *51*, 1884.
[9] L. Sham, M. Schlüter, *Phys. Rev. Lett.* **1983**, *51*, 1888.
[10] J. Klimeš, A. Michaelides, *J. Chem. Phys.* **2012**, *137*, 120901.
[11] L. Kronik, A. Tkatchenko, *Acc. Chem. Res.* **2014**, *47*, 3208.
[12] W. A. Al-Saidi, V. K. Voora, K. D. Jordan, *J. Chem. Theory Comput.* **2012**, *8*, 1503–1513.
[13] T. Bučko, S. Lebègue, J. Hafner, J. Ángyán, *Phys. Rev. B* **2013**, *87*, 064110.
[14] A. Tkatchenko, M. Scheffler, *Phys. Rev. Lett.* **2009**, *102*, 073005.
[15] J. Heyd, G. E. Scuseria, M. Ernzerhof, *J. Chem. Phys.* **2003**, *118*, 8207.
[16] J. Heyd, G. E. Scuseria, M. Ernzerhof, *J. Chem. Phys.* **2006**, *124*, 219906.
[17] A. Tkatchenko, R. A. DiStasio, R. Car, M. Scheffler, *Phys. Rev. Lett.* **2012**, *108*, 236402.
